# Anchors Away: The Critical Role of Membrane Bound Molecules in Regulating Stem Cell Symmetry

**DOI:** 10.1097/HS9.0000000000000678

**Published:** 2021-12-30

**Authors:** David G. Kent

**Affiliations:** Department of Biology, York Biomedical Research Institute, University of York, United Kingdom.

One of the most captivating model systems in all of stem cell niche biology is the Drosophila germ stem cell niche where the simplicity is just beautiful. There is a defined space for stem cells, and they have defined supporting neighbour cells (cap cells) to such an extent that creating more supporting cells results in more stem cells and removing supporting cells obliterates them.^1^ The relationship is such that the germline stem cells divide asymmetrically with respect to these cap cells and produce one daughter stem cell that stays in the niche and one that leaves and differentiates—textbook asymmetric maintenance self-renewal. When we think about the hematopoietic stem cell (HSC) system in mammals and try to reconcile this concept of physical location with a sort of “one in, one out” type system, it quickly becomes mired in the decades long battle of which cell types and molecules comprise the adult bone marrow (BM) niche for HSCs. One of the few things these papers all seem to agree on is the involvement of stem cell factor (SCF) and a recent paper from Keyue Shen’s lab highlights the critical physical role of membrane bound SCF in regulating HSC polarity.^2^

SCF has been the cardinal HSC niche molecule since a series of experiments using mice mutant at the *W* (c-Kit) and *Sl* (SCF) loci, which showed that *W* mutant HSCs had an intrinsic defect and were less functional in colony assays and transplantation experiments whereas mutations in the *Sl* locus imparted a microenvironmental defect where HSCs could not function as well when transplanted into mice. BM from *Sl* mutants, however, could be transplanted into new mice with a normal microenvironment and they would perform normally, clearly demonstrating that the effect of *Sl* mutations were in nonhematopoietic cells that supported HSCs (reviewed in Kent et al^3^). Following the identification of SCF as the gene product of the *Sl* locus responsible for this difference, it was also later discovered that SCF was unique from many hematopoietic cytokines in that it had both a membrane bound form and a soluble form. Further experiments using the Sl/Sl^d^ mouse (which only expresses the soluble form) showed that the membrane bound form was critical for maintaining the number and function of HSCs.^4^

Fast forward 25 years and we still do not really have a formalised “one in, one out” set of rules for the hematopoietic system, and without a strong physical orientation it becomes quite difficult to ascribe any function to the orientation of HSCs with respect to mother and daughter cells. This is where the paper of Hao et al, comes in. Using soluble lipid bilayers (SLBs) to permit lateral mobility of proteins alongside the ability to cluster, they initiated a series of experiments to determine whether SCF-mediated HSC cell polarity. Their experiments showed that SCF alone could induce protrusions from HSCs, but only in the presence of VCAM-1 could they do so in a highly polarised fashion. Moreover, when soluble SCF was added to the mix, it competitively disrupted the process of establishing such strong polarity. Following this, they undertook a series of experiments to explore the impact of polarity on the adhesion properties of HSCs and showed that the combination of VCAM-1 and SCF also altered HSC adhesion to the SLB, implying that these molecules could serve the same role in increasing an HSC’s ability to lodge—and stay lodged—in the BM niche.

These studies are important to consider in the context of recent work that has shown that the self-renewal capacity of HSCs can be linked to the asymmetric (or symmetric) partitioning of various cellular organelles. Beautiful work from Loeffler et al^5,6^ has shown that asymmetric lysosome and mitochondria segregation in mouse and human HSCs can confer different metabolic and translational activity to each daughter cell. We are entering an age where the tools for bioengineering a niche and for assessing HSCs directly in vivo put us on the cusp of finally being able to experimentally address many of these core questions that have been sitting around for decades. This sets the stage for some exciting work in the stem cell niche and modulation of stem cell fate through advanced cellular and molecular tools where we can now start to imagine harnessing cell polarity and asymmetric distribution of cellular components to understand aberrant self-renewal in haematological malignancies (i.e., is polarity disrupted or rendered unnecessary?) and to achieve large scale directed differentiation outside the body for therapeutic purposes.

## DISCLOSURES

The author has no conflicts of interest to disclose.

## References

[1] Lin H. The stem-cell niche theory: lessons from flies. Nat Rev Genet. 2002;3:931–940. 12459723 10.1038/nrg952

[2] Hao J Zhou H Nemes K. Membrane-bound SCF and VCAM-1 synergistically regulate the morphology of hematopoietic stem cells. J Cell Biol. 2021;220:e202010118. 34402812 10.1083/jcb.202010118 PMC8374872

[3] Kent D Copley M Benz C. Regulation of hematopoietic stem cells by the steel factor/KIT signaling pathway. Clin Cancer Res. 2008;14:1926–1930. 18381929 10.1158/1078-0432.CCR-07-5134

[4] Barker JE. Early transplantation to a normal microenvironment prevents the development of Steel hematopoietic stem cell defects. Exp Hematol. 1997;25:542–547. 9197334

[5] Loeffler D Schneiter F Wang W. Asymmetric organelle inheritance predicts human blood stem cell fate. Blood. 2021 July 27. [Epub ahead of print]. 10.1182/blood.2020009778 34314497

[6] Loeffler D Wehling A Schneiter F. Asymmetric lysosome inheritance predicts activation of haematopoietic stem cells. Nature. 2019;573:426–429. 31485073 10.1038/s41586-019-1531-6

